# The Role of Oxytocin and Oxytocin Gene Receptor Methylation During Withdrawal Therapy in Males With Alcohol Use Disorder

**DOI:** 10.1111/adb.70060

**Published:** 2025-07-14

**Authors:** Phileas J. Proskynitopoulos, Alissa F. Haarmeyer, Stefan Bleich, Helge Frieling, Thomas Hillemacher, Alexander Glahn, Mathias Rhein

**Affiliations:** ^1^ Department of Psychiatry, Social Psychiatry and Psychotherapy Hannover Medical School Hannover Germany; ^2^ Laboratory for Molecular Neuroscience Hannover Medical School Hannover Germany; ^3^ Department of Psychiatry and Psychotherapy Paracelsus Medical University Nuremberg Germany

**Keywords:** alcohol use disorder, DNA methylation, epigenetics, oxytocin, substance use disorder

## Abstract

Oxytocin is a promising therapeutic target in the treatment of alcohol use disorder (AUD). However, many studies report contradicting evidence regarding its effect on drug craving, relapse risk and withdrawal symptoms. Epigenetic regulation of the oxytocin and oxytocin receptor (OXTR) gene is altered in several mental disorders and influences social behaviour, often depending on the underlying sex. Evidence suggests that altered promoter methylation could result in oxytocin and OXTR expression differences, thereby possibly influencing drug craving and relapse risk. It is unclear whether promoter methylation changes throughout alcohol withdrawal and is linked to craving and withdrawal symptoms. In this exploratory study, we investigated the effect of 2‐week alcohol withdrawal therapy in 99 males on methylation levels (oxytocin and OXTR) compared with 31 healthy controls. We found significantly higher mean methylation values of the OXTR gene in controls than patients across withdrawal (*p* < 0.001). Regarding oxytocin, we found no differences in mean methylation in healthy controls compared with patients. Across withdrawal, mean methylation decreased in both genes. Fitting a mixed linear model, craving and withdrawal symptoms were associated with changes in methylation levels of the oxytocin gene (*p* < 0.001), which was also true for the OXTR gene when considering age and smoking as additional covariates. Our study is the first to report an association between AUD, oxytocin and OXTR gene methylation. Methylation of the OXTR gene is reduced in AUD compared with healthy controls, with OT gene methylation linked to craving and withdrawal severity. Our results suggest that investigations of oxytocin as a therapeutic agent need to consider epigenetic regulation of its receptor and gene as a mechanism that could influence oxytocin's effect on craving and withdrawal symptoms.

## Graphical abstract

This study investigates the effect of alcohol withdrawal therapy on promoter methylation of the oxytocin and oxytocin receptor gene. We found significantly higher mean methylation values of the receptor gene in controls compared with patients across withdrawal and no differences in mean methylation of the oxytocin gene. Our results suggest that investigations of oxytocin as a therapeutic agent need to consider epigenetic regulation of its receptor and gene.

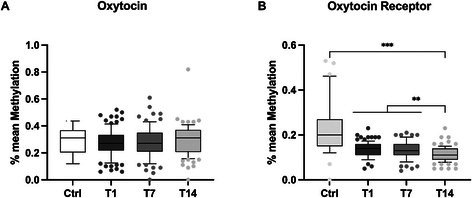

## 1 Introduction

In recent years, new therapeutic targets have been studied for treating alcohol use disorder (AUD), one being the neuropeptide oxytocin (OT). As a hormone, it affects the physiological stress reaction and the effect of other hormones throughout the central nervous system. It is well established that oxytocinergic neurons connect to several brain areas involved in the neurocircuitry model of addiction [1], such as the limbic system and Ncl. accumbens [2, 3, 4]. A recent review summarizes the evidence showing that in both males and females, alcohol seems to inhibit OT release and production while, at the same time, the effects on the OT system seem to change with repeated exposure to alcohol [5]. In animals, studies demonstrated that intracerebroventricular administration of OT could completely block ethanol‐induced dopamine release within the Ncl. accumbens of rats [6] and that intraperitoneal OT reduced alcohol consumption in prairie voles [7]. More recently, OT reduced alcohol cue reactivity in alcohol‐dependent rats and humans [8]. Interestingly, oxytocin receptors (OXTRs) were upregulated in brain tissues of alcohol‐dependent rats and deceased persons suffering from AUD, primarily in the frontal and striatal areas [8]. In a clinical pilot study using functional magnetic resonance imaging in male heavy social drinkers, intranasal OT could decrease neuronal cue reactivity in brain networks similar to those detected in alcohol‐dependent rats and humans with increased OXTR expression [8].

However, also contradicting evidence exists concerning its effect on fundamental symptoms of AUD, such as withdrawal and craving. While one study has shown that intranasal OT may block alcohol withdrawal in human subjects [9], another study reported that intranasal OT does not reduce oxazepam dosages during a 3‐day withdrawal course [10]. Regarding craving, in one randomized double‐blind and placebo‐controlled trial, intranasal OT did not mitigate alcohol craving or aggression [11].

The OXTR network in the rodent and human brain is substantially influenced by the underlying sex [12], with OT having profound effects on complex behaviour such as parenting [13] or trust [14, 15]. Hansson and Spanagel [16] commented on their previous work [8] and presented evidence that decreased OT expression and increased OXTR binding sites are only apparent in males but not found in females [16]. Thus, it is fair to argue that OT exerts sex‐specific effects in the context of AUD.

In summary, the effect of OT administration is related to its receptor expression and appears to be differently regulated in females and males. Therefore, to find potential explanations for its sex‐specific and contradictory effects, it is essential to study neurobiological and physiological factors that could influence oxytocin's impact on neurocircuits of addiction. Interestingly, the effect of OT and its potentially positive influence on craving and relapse is linked directly to the expression of its receptor. OXTR expression is regulated partly via promoter methylation [17]. Furthermore, genetic variations, such as single nucleotide polymorphisms of the OXTR, affect impulsivity [18], a significant risk for addictive behaviour. Apart from that, one study has shown that carriers with a specific rs53576 OXTR genotype may present a greater biological sensitivity and stress reactivity in the context of environmental adaptation [19]. Thus, we believe that differences and changes in promoter methylation could result in differences in the expression of both OT and its receptor, thereby influencing its effect on substance craving in the human brain and potentially the effect of intranasal OT on craving and withdrawal symptoms.

To our knowledge, no study has investigated differences in OXTR and OT promoter methylation in males with AUD. In the present study, we hypothesize that (1) OT and OXTR receptor methylation differs in patients suffering from AUD compared with healthy controls and (2) changes throughout cessation treatment. Furthermore, we hypothesize that specific promoter variations, such as mean methylation or single CpG methylation levels, are associated with psychometric measurements such as drug craving and withdrawal symptoms.

## 2 Subjects and Methods

### 2.1 Participants

The current exploratory study is part of a large prospective research project, ‘Studies in Neuroendocrinology and Neurogenetics in Alcoholism (NENA)’. The local Ethics Committee of the University of Erlangen‐Nuremberg approved the original study. The Ethics Committee at Hannover Medical School approved the described investigation (approval number 10399_BO_K_2022). The investigation followed the Declaration of Helsinki. Before enrolment, each participant gave written informed consent to participate in the study. All patients included suffered from alcohol dependence according to DSM‐IV and ICD‐10. The study and analyses were not pre‐registered. Clinical assessment was performed by a trained physician following a standardized protocol following the diagnosis criteria of ICD‐10 and DSM‐IV. Patients with comorbidities concerning mental disorders, other substances of abuse apart from alcohol or nicotine, the existence of severe somatic pathologies, known HPA‐axis, or thyroid diseases were excluded from this study. After enrolment, all patients underwent a detailed physical examination by a professional (clinician), routine laboratory testing, and urine drug screening. As a control group, we included males who were negative for alcohol abuse, alcohol dependence, or other mental or somatic disorders according to ICD‐10 or DSM‐IV. Fasting blood samples were drawn between 8:00 and 10:00 AM. Then, all blood samples were centrifuged and stored at −80°C immediately after collection. The methylation rate of the OT and OXTR receptor genes has been investigated during three time points, namely on Day 1, Day 7 and Day 14 of alcohol withdrawal. Blood alcohol concentration (BAC) was measured at admission by using an enzymatic assay (Roche/Hitachi Ethyl Alcohol, Mannheim/Germany, 904/911: ACN 270). Besides, the extent of alcohol craving was obtained at admission and during detoxification treatment using the Obsessive‐Compulsive Drinking Scale (OCDS), which provides a well‐validated and reliable assessment [20]. For the assessment of withdrawal severity, we used the Clinical Institute Withdrawal Assessment for Alcohol Scale (CIWA) [21]. Severity of alcohol dependence was assessed with the ‘Scale for the Assessment of Alcohol Dependence Severity’ (in German ‘Skala zur Erfassung der Schwere der Alkoholabhängigkeit’, SESA) [22]. Depressive symptoms were measured with the Beck Depression Inventory (BDI) [23] and State–Trait Anxiety Inventory (STAI) [24], respectively.

### 2.2 DNA Isolation and Bisulfite Conversion

Extraction and clean‐up of genomic DNA from blood were done using the NucleoMag Blood 200‐μL DNA Kit (Macherey‐Nagel, Düren, Germany). The bisulfite conversion of the acquired DNA samples was done using the EpiTect 96 Bisulfite Kit (QIAGEN, Hilden, Germany) for OXTR and EZ96 Magprep Bisulfite Kit (Zymo Research Europe, Freiburg, Germany) for OT following the manufacturer's protocol.

### 2.3 Primer Design

All primers were designed manually to bisulfite‐converted regions of the respective genes using the program Geneious (Biomatters, Auckland, New Zealand). All fragments covered the proximal promoter region of the respective gene. For our analysis of the OT gene, we designed and used the forward primer F1 TTTGTTTTATTTTAGTGGTTTAGGTTAT and reverse primer R1 CTCAACTCCTAAAATTCTCAAA. The total fragment size was 512 bp. For OXTR, we designed the forward primer F1 GGTATTTTATTTTTTGTGTTTAGATTAT and reverse primer ACTCACTACAAACTCTACCTCC. The total fragment size was 379 bp.

We used the primer analysis tool Netprimer (http://www.premierbiosoft.com, accessed on 01.8.2022) to check for the presence of secondary structures (e.g., hairpins and self‐primer). Melting temperatures were also retrieved from Netprimer analysis. The resulting primers were ordered from Metabion (Metabion, Steinkirchen, Germany).

### 2.4 Amplification of the Bisulfite‐Converted Target Sequences

Polymerase chain reactions (PCRs) were performed in a C1000 Thermal Cycler (BIO‐RAD, Hercules, CA, USA). The target sequences of the purified bisulfite‐converted DNA were amplified following a standard PCR protocol. The reaction components used were as follows: 0.4 μL (20 μmol) forward primer (F1 for OXTR, F1 for OT), 0.4 μL (20 μmol) reverse primer (R1 for OXTR, R1 for OT), 1 μL of DNA, 3.2 μL of H_2_O and 5‐μL HotStarTaq Master Mix Kit (Qiagen, Hilden, Germany). The bisulfite primer amplification temperature was set at 53°C (OXTR)/57°C (OT) for the polymerase chain reaction. Amplification products were purified using the Agencourt AMPure XP magnetic beads (Beckman Coulter).

### 2.5 Sequencing

Sequencing PCR for the target fragment was performed using a BigDye Terminator v3.1 Cycle Sequencing Kit (Applied Biosystems, Foster City, CA, USA) and an Applied Biosystems/HITACHI 3500xl Genetic Analyzer (Applied Biosystems) according to the manufacturer's instructions. The bisulfite primers F1_OXTR and R1_OT were used in 5‐pM concentrations for the PCR reaction of the respective genes. The sequencing PCR products were purified using the Agencourt CleanSeq XP magnetic beads (Beckman Coulter) and then used for sequencing. Electropherograms and sequences detected by the Genetic Analyzer were processed using the specialized epigenetic sequencing methylation analysis software to determine the methylation rates for every CpG locus.

### 2.6 Analysis of Methylation Rates

We used the Epigenetic Sequencing Methylation Software (ESME) software package to determine methylation rates. After normalization to the highest fluorophore intensity, ESME aligns the generated and reference sequences for comparing methylation at each CpG site. CpG islands were then labelled in relation to the location from the first base pair of exon 1 (i.e., CpGp134 would be 134 bp in the 5′ direction from the exon 1 [p = plus], and CpGm112 would be 112 bp in the 3′ direction from the exon 1 [m = minus]). We calculated quantitative methylation for each site per subject (proportion of cytosine and thymine normalized peak values) [25].

### 2.7 Statistical Analysis

We used the Statistical Package for Social Sciences Versions 27, 28 and 29 (SPSS, IBM, Armonk, NY, USA) and GraphPad Prism version 7 and 9 (San Diego, CA, USA) for all statistical analyses. The latter was further used for data illustration.

As a standard procedure in our laboratory, we performed quality control of our methylation analysis. We included only those CpGs with less than 5% missing values for each gene. Then, we excluded each sample with more than 5% missing values. After applying these criteria, the total number of CpGs for OT were 26 and OXTR were 36.

From visual inspection, normality was present in the methylation data of the OXTR but not OT gene. For comparative reasons, we decided to perform nonparametric tests to assess any differences between healthy controls and patients and changes throughout withdrawal. In detail, to test for differences in mean methylation levels scores across the withdrawal period, we used the nonparametric Friedman's test for dependent samples. To test for differences between healthy controls and the different timepoints, we used the Kruskal–Wallis test or Whitney *U* test. The Bonferroni correction was applied to correct for multiple testing.

To assess changes in gene methylation throughout the cessation period and the effect of craving and withdrawal on methylation levels, we fitted a mixed linear model (MLM). In this model, mean methylation was set as the dependent variable, while CpG position as well as time points were set as factors while withdrawal severity and craving were set as covariates. The resulting estimated marginal means (EMMs) were used to compare methylation values corrected for the influence of the factors. We used post hoc tests to compare mean methylation at the different time points (Bonferroni's method) and Bonferroni's method to correct for multiple testing. To compare the effect of sex and timepoint on the EMMs we fitted a one‐way ANOVA with post hoc tests and correction for multiple testing (Tukey's method). We also included age and amount of cigarettes smoked into the mixed modelling and correlated them with mean methylation to estimate the confounding effect of these influential epigenetic modifiers.

## 3 Results

### 3.1 Demographics

The study population consists of 99 patients suffering from AUD and 31 healthy male controls (Table 1). The study population is already described in detail elsewhere [26].

**TABLE 1:** Demographics.

	Controls			Patients		
	Mean	Standard deviation	Valid *N*	Mean	Standard deviation	Valid *N*
Age	44.06	11.46	31	43.15	8.24	99
School years	12	1	31	9	1	99
Smokers (*n*)	22			81		
Ex‐smokers (*n*)	0			6		
Cigarettes per day			0	23	10	78
BDI	3	3	29	17	9	98
STAI				48	12	98
Daily intake in g				190.65	82.24	94
SESA				48.28	18.51	97
OCDS			0	19.76	6.90	99
CIWA			0	15.46	4.03	99

Abbreviations: BDI = Beck Depression Inventory; CIWA = Clinical Institute Withdrawal Assessment for Alcohol Scale; OCDS = Obsessive‐Compulsive Drinking Scale; SESA = Scale for the Assessment of Alcohol Dependency Severity ‘Skala zur Erfassung der Schwere der Alkoholabhängigkeit’; STAI = State–Trait Anxiety Inventory.

### 3.2 Methylation in Healthy Controls vs. Subjects (Baseline)

**FIGURE 1:**
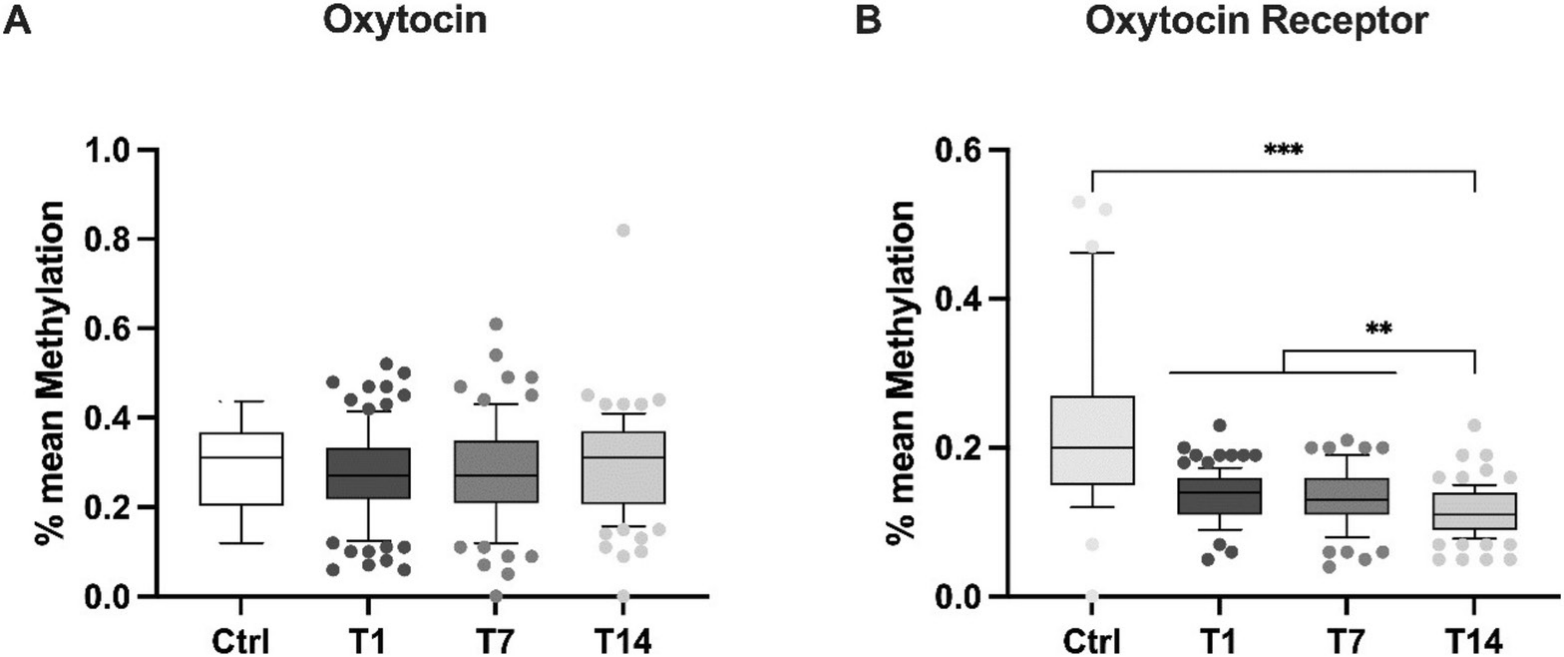
Boxplots displaying levels of mean methylation (percentage of CpG islands methylated in the studied region) of the oxytocin (A) and oxytocin receptor gene (B) in healthy controls and during withdrwaral therapy. Significant differences, as calculated with nonparametric tests, are indicated by asterisks. *p < 0.05, **p < 0.01, ***p < 0.001.

After quality control, 26 CpGs for the OT gene and 36 for OXTR were analysed (Figure 1). Because there was no difference in mean methylation across the OT gene in controls compared with patients across all time points, we individually plotted the methylation values of each CpG in the OT promoter fragment. From visual inspection, we identified a cluster ranging from base 42 to 151 across 11 CpGs (Cluster 11) (Figure S1).

Because normality was absent in the methylation data of the OT gene, we used the Friedman test to compare mean methylation across all CpGs and the cluster. There was no change in mean methylation across withdrawal when using non‐parametric tests. Cluster 11 mean methylation changed significantly during cessation (Friedman statistic = 7.327, *p* = 0.0256).

Regarding the OXTR gene, mean methylation was significantly higher in healthy controls compared with all‐time points (t14: *χ* ^2^(3) = 137.895, *p* < 0.001; t7: *χ* ^2^(3) = 89.325, *p* < 0.001; t0: *χ* ^2^(3) = 92.602, *p* < 0.001). Furthermore, mean methylation decreased significantly during withdrawal therapy (Friedman statistic = 17.02, *p* = 0.0002; Figure 1).

### 3.3 Effect of Timepoint, Withdrawal Severity and Craving on Gene Methylation

In our model, CpG position, timepoint, withdrawal severity score and the interaction between timepoint and craving, timepoint and withdrawal severity and timepoint, craving and withdrawal severity had a significant fixed effect on OT gene methylation (Table 2).

**TABLE 2:** Type III tests of fixed effects for the dependent variable methylation (oxytocin).

Source	Numerator df	Denominator df	*F*	Sig.
Intercept	1	6801	348.72	< 0.001
CpG position	25	6801	352.4	< 0.001
Timepoint	2	6801	54.804	< 0.001
OCDS	1	6801	1.08	0.299
CIWA	1	6801	11.911	< 0.001
Timepoint * OCDS	2	6801	36.77	< 0.001
Timepoint * CIWA	2	6801	49.226	< 0.001
OCDS * CIWA	1	6801	2.336	0.126
Timepoint * OCDS * CIWA	2	6801	34.819	< 0.001

Abbreviations: CIWA = Clinical Institute Withdrawal Assessment for Alcohol Scale; OCDS = Obsessive‐Compulsive Drinking Scale.

**FIGURE 2:**
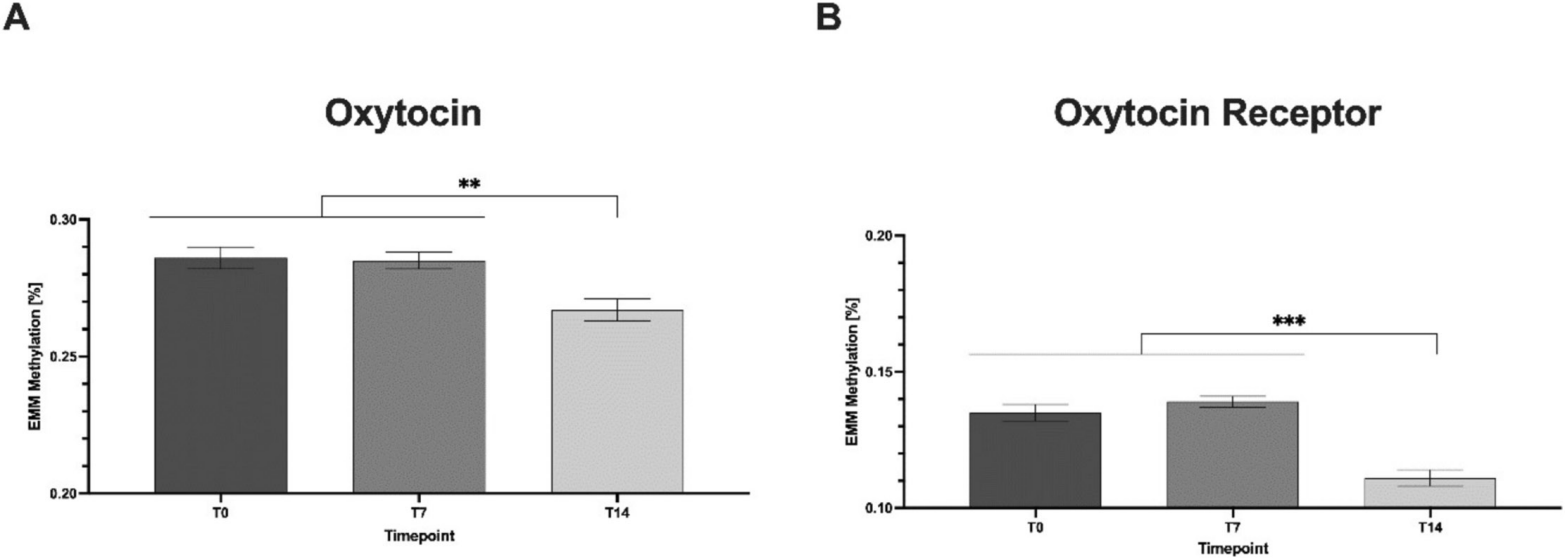
Modelled changes in mean methylation of the oxytocin (A) and oxytocin receptor gene (B) during withdrawal therapy. Mean values are the estimated marginal means (EMMs) reported by the mixed linear model. The EMMs were then compared in a one‐way ANOVA. Each bar represents the mean ± standard error of the mean. Significant differences are indicated by asterisks. *p < 0.05, **p < 0.01, ***p < 0.001.

Post hoc tests (Tukey) showed that mean methylation (based on expected marginal means, EMM; Figure 2) was significantly lower at t14 (mean = 0.267) compared with t0 (mean = 0.286) and t7 (mean = 0.285) (mean difference (MD) = 0.019, *p* = 0.004, 95% CI = [0.005–0.33]; MD = 0.017, *p* = 0.004, 95% CI = [0.004–0.30], respectively).

We also fitted a mixed linear model to analyse the effect of psychometrics and time points on OXTR methylation levels. In this model, only CpG‐position had a significant effect on mean methylation levels (Table 3). Post hoc tests (Tukey) showed that mean methylation (based on EMM; Figure 2) was significantly lower at t14 (mean = 0.111) compared with t0 (mean = 0.135) and t7 (mean = 0.139) (MD = 0.024, *p* ≤ 0.001, 95% CI = [0.015–0.33]; MD = 0.028, *p* ≤ 0.001, 95% CI = [0.020–0.36], respectively).

**TABLE 3:** Type III tests of fixed effects for the dependent variable methylation (oxytocin receptor).

Source	Numerator df	Denominator df	*F*	Sig.
Intercept	1	9585.000	133.711	< 0.001
CpG_pos	35	9585.000	93.786	< 0.001
Timepoint	2	9585.000	0.255	0.775
OCDS_total	1	9585.000	1.313	0.252
CIWA	1	9585.000	1.294	0.255
Timepoint * OCDS_total	2	9585	1.379	0.252
Timepoint * CIWA	2	9585.000	1.614	0.199
OCDS_total * CIWA	1	9585.000	0.177	0.674
Timepoint * OCDS_total * CIWA	2	9585.000	0.191	0.826

Abbreviations: CIWA = Clinical Institute Withdrawal Assessment for Alcohol Scale; OCDS = Obsessive‐Compulsive Drinking Scale.

Controlling for confounders, we modelled psychometry against age and amount of smoked cigarettes for both genes. Here, in the OXTR receptor, it shows no singular relevance of age and cigarettes on methylation, but combinatorial effects of timepoint and age (*F* = 3.085, *p* = 0.046), CIWA (*F* = 3.762, *p* = 0.023) and OCDS (*F* = 8.482, *p* < 0.01) reveal subtle influences on overall methylation. OT methylation shows significance for the single fixed effects of age (*F* = 4.685, *p* = 0.03) and amount of cigarettes (*F* = 43.968, *p* < 0.001), as well as for most combinatorial effects, which are driven by this main confounder (see Supporting Information S2). Because we did not observe a significant difference in mean methylation of the OT promoter in controls compared with patients, we conducted a post hoc power analysis. When implementing mean values and the number of subjects at an alpha level of 0.05, the post hoc power for the oxytocin promoter was 27.5%, while for the receptor it was 99.2% (see Supporting Information S3).

## 4 Discussion

In this study, we hypothesize that OT and OXTR receptor methylation differs in patients suffering from AUD compared with healthy controls and would change throughout withdrawal treatment. Furthermore, we hypothesized that specific promoter variations, such as mean methylation or single CpG methylation levels, are associated with psychometric measurements such as alcohol craving and withdrawal symptoms. We found significantly higher mean methylation values of the OXTR gene in controls compared with patients across the withdrawal period. Regarding the OT gene, we found no difference in mean methylation between healthy controls and patients. Across withdrawal, depending on the statistical analysis used, mean methylation decreased in both genes.

The observed decrease in methylation values of both the receptor and its ligand across the withdrawal period is counterintuitive when considering the general physiological mechanisms. Usually, an increase in a ligand normally leads to a reduction in the expression of its respective receptor for the synapse to remain excitable. Because gene methylation is an epigenetic mechanism that usually diminishes gene expression, our results suggest a simultaneous increase in OT and OXTR gene expression during withdrawal therapy. Several studies have shown that alcohol has a direct effect on oxytocin and OXTR expression [5]. For example, oxytocin peptide mRNA was significantly higher in the prefrontal cortex of subjects with AUD compared with healthy controls [27], with most studies suggesting an inhibitory effect of alcohol on oxytocin levels [5]. In one study, the authors were able to show an upregulation of OXTR in both rats and deceased persons with AUD, most likely due to reduced OT expression in hypothalamic nuclei [8]. Assuming that gene methylation in blood cells is related to gene methylation in brain tissue, our results align with the findings of Hansson et al. [8]. We found higher methylation values in healthy controls, suggesting a downregulation of OXTR. During withdrawal, however, OT methylation values and OXTR methylation values both decreased. Of note, OT methylation only decreased in the mixed linear model and when looking at individual CpGs. Also, the net amount of change in methylation for OT also was considerably smaller than for the OXTR promoter, which offers much more robust mean methylation differences, both concerning controls vs. patients as well as during withdrawal. These results suggest a complex interplay between both genes when alcohol is withdrawn. As Ryabinin and Fulenwider point out, the effect of alcohol on the oxytocin system depends on a complex interplay between factors such as experimental paradigm, sex, genotype and species [5]. This may also be due to the comparably distant regulatory role alcohol plays for the oxytocin homeostasis.

In addition, because we measured gene methylation in peripheral whole blood, it is more likely that changes in gene methylation translate into plasma changes in the respective genes rather than changes in central, that is, brain tissue levels. Even though several studies have shown that alcohol tends to inhibit the stimulus‐evoked release of OT, Ryabinin and Fulenwider conclude in their review that tolerance in the peripheral component of the OT system can prevent such an effect after repeated alcohol exposures [5]. On the other hand, as alcohol levels tend to be comparable upon distribution of this amphiphilic cellular toxin throughout the body, the likelihood of cellular regulation in different tissues is higher compared with other drugs and stimuli [28]. Thus, it is fair to argue that our study adds relevant findings to the evidence of the complex interplay between alcohol and the oxytocin system.

Our research group has previously investigated gene methylation of vasopressin during alcohol withdrawal therapy in the study sample investigated here [29]. Oxytocin and vasopressin are both secreted in the neurohypophysis and structurally very similar [2, 30] while also being able to bind to the respective opposite receptor [31, 32]. Nonetheless, their function and physiological effects on various human behaviours seem to differ across species and in the male and female sex [33, 34, 35, 36]. In the study by Glahn et al. [29], vasopressin gene methylation was significantly lower in the AUD patients on Days 7 and 14 than in healthy controls. In the present study, mean methylation of the OT gene was not different in healthy controls compared with patients. In our model, however, OT gene methylation decreased during withdrawal treatment. Taken together, epigenetic regulation of both the AVP and OT genes seems to differ in AUD, with both seeming to be hypomethylated throughout withdrawal in male AUD patients.

One explanation for the decrease in methylation values over withdrawal could be the effect of stress on leukocyte expression in peripheral blood [37]. As Kumsta and colleagues point out, changes in the expression of mononuclear cells into the peripheral blood during stress could affect overall methylation values [17]. As alcohol has both a toxic effect on blood cell production [38] and an immediate effect on leukocyte function [39], the alcohol levels at the beginning of the withdrawal period and the severity of bone marrow insufficiency could directly influence methylation levels.

In our study, withdrawal severity, as assessed by the CIWA, significantly associated with OT but not OXTR gene methylation. This link could be explained by the neurochemical mechanisms of alcohol withdrawal, leading to central nervous system hyperexcitability and an increase in the activity of the autonomous nervous system [40] and activation of the hypothalamus–pituitary–adrenal axis (HPA‐axis) [41]. With stress affecting the composition of the circulating leukocytes [37] and CIWA scores being associated with the severity of central nervous system hyperexcitability, the association of withdrawal severity and methylation could indeed be explained by this relationship.

Of course, the final expression and effect on physiology during stress comprises many more rate‐limiting regulatory events than mere methylation. Also, we only found an association between withdrawal symptoms in one of the two genes investigated, suggesting that the relationship between OT gene methylation and craving as well as withdrawal symptoms is also affected by other factors. More importantly, HPA‐axis activity during withdrawal is likely to partly explain craving scores and withdrawal severity and the relationship between craving, withdrawal symptoms and OT gene methylation. It is well established that stress and HPA‐axis activation are linked to relapse risk, withdrawal symptoms and craving [41, 42, 43]. In our study, OT receptor methylation was linked to alcohol craving and withdrawal symptoms potentially leading to HPA‐axis activation. Observations by others display the oxytocin system as an antistress system and that both are antagonistic in nature while influencing each other [44]. As a hormone, oxytocin affects the physiological stress reaction and the effect of several other hormones throughout the central nervous system. As Üvnas‐Moberg et al. point out in their review, OT exerts strong inhibitory effects on theHPA‐axis in three different ways: It decreases the secretion of CRH, inhibits ACTH secretion from the anterior pituitary and is able to decrease the release of cortisol by a direct mechanism in the adrenals [44]. CRH is one of the main hormones in the HPA axis: Following its secretion, adrenocorticotropin (ACTH) is produced, resulting in cortisol secretion and the physiological stress reaction. Furthermore, CRH has a central role in the pathogenesis of AUD. Chronic alcohol intake results in changes in CRH activity and in different brain areas [45]. Acute drug intake results in the activation of the HPA axis and an increase in the secretion of CRH and ACTH into the bloodstream while also being secreted increasingly in the amygdala. During withdrawal, systemic CRH release is inhibited. In the amygdala, however, CRH secretion increases [46]. More interestingly, one study has shown that higher CRH receptor expression in specific areas of the amygdala increases vulnerability to relapse [47]. Thus, in summary, the association between craving, withdrawal symptoms and OT methylation could, to some extent, be explained by its function as an antistress system counterbalancing the HPA‐axis while also being affected by acute and chronic alcohol intake.

Because we report a clear difference between OXTR methylation in AUD compared with healthy controls, one may raise the question of whether this difference is a result of alcohol use or a predisposition to it. Our study design, however, does not allow any conclusions about the causal relationship between OXTR methylation and AUD. While the changes in OXTR methylation over withdrawal suggest a direct effect of alcohol use, it is unclear to what extent values would regress toward those found in healthy controls. Notably, values decreased during withdrawal, while controls had significantly higher values initially. Future studies would need to clarify whether these modifications predate substance exposure or whether they appear only after prolonged drug intake [48].

With oxytocin being increasingly used as a therapeutic agent and target in substance use disorders, our study's findings could have some clinical and therapeutic implications. In detail, due to the robust difference found in OXTR gene methylation between males with AUD and healthy controls, future studies could investigate its use as a biomarker for AUD. Furthermore, it could be taken into account as a factor when testing the effect of intranasal oxytocin on drug craving. However, translating experimental findings in epigenetics research is very difficult [48, 49]. In order to understand those difficulties in greater detail, it is important to discuss the study's limitations.

### 4.1 Limitations

Naturally, the study has several limitations, some of which we have already discussed in another study on oxytocin and OXTR methylation in tobacco use disorder [50]. Epigenetic research is fraught with challenges, such as tissue specificity, reversibility of epigenetic marks and technical variability. While being matched for sex, we did not match patients and controls for other factors such as age [51] and smoking status [52], which undoubtedly affect gene methylation. In our study, smoking (as assessed with average cigarettes per day) and age affected oxytocin but not OXTR promoter methylation. Furthermore, we did not observe an association with CIWA scores and methylation but with OCDS. However, regarding OXTR promoter methylation, the inclusion of age and smoking revealed an association of methylation levels with the factors timepoint * OCDS as well as timepoint * CIWA. Of note, this was also found in the OT model with and without the mentioned factors smoking and age. This suggests an association between psychometrics and methylation depending on the timepoint measured. Furthermore, our post hoc power analysis suggests that a larger sample size and matching for factors such as age and smoking status would be needed to identify a potential difference in mean methylation of the OT gene. OXTR promoter methylation, on the other hand, seems to be less influenced by the mentioned factors age and smoking and more by the main differentiator between the groups, namely, alcohol consumption. However, the fact that we only observe an interaction between timepoint and psychometric variables in OXTR promoter after inclusion of age and smoking reveals that both variables play a major role in the methylation levels.

Regarding sex differences, an increasing number of studies have presented evidence for a sex‐specific effect of intranasal oxytocin on human behaviour [14, 53] and sex‐specific differences in the oxytocin system [33, 34, 35, 36]. As highlighted in the introduction, sex‐specific differences in OXTR function and addiction behaviours are well established. Thus, the absence of female participants limits the broader applicability of our findings. Regarding tissue specificity, we only measured gene methylation in peripheral whole blood; therefore, our results do not allow any conclusions about changes in methylation levels in the brain or other specific tissues. Of note, while some studies describe an association between OXTR promoter methylation and OXTR expression in brain tissue [54, 55], one thorough review discusses why it is unclear of and how peripheral measurements reliably mirror neural processes [56, 57]. As discussed above, studies on the effect of alcohol withdrawal on methylation levels need to consider the direct effect of alcohol and withdrawal severity on peripheral blood cells. In the present study, we did not control the alcohol level or the autonomic system activation when the blood was drawn. With respect to the reversibility of epigenetic marks, our results suggest a certain reversibility over time because methylation values changed significantly over withdrawal. However, complete reversibility cannot be concluded from our data due to the study design (such as the short observational period).

Regarding the experimental/technical methods used in this study, namely, Sanger sequencing, it is important to point out that modern sequencing methods, such as target enrichment sequencing and Oxford Nanopore Technology Sequencing, have methodic advantages compared with Sanger [58]. On the other hand, Sanger sequencing is the gold standard for comparison and investigation of specific genetic loci. Concerning its accuracy for measuring single promoter regions, our research group has shown that it achieves comparable results with ONT‐S when analysing promoter methylation [59]. Nonetheless, future studies investigating the Oxytocin system in addiction and across the sexes should use more modern sequencing techniques such as ONT‐S. One methodological limitation of the present study is the difference in group sizes between controls and patients. In detail, this group difference and nonparametric testing increase the probability of a type two error, that is, not rejecting the null hypothesis even though the alternative hypothesis is true. Future studies would need more balanced samples to minimize such effects on reliability.

### 4.2 Conclusion

In summary, our study is the first to report an association between AUD and OT and OXTR gene methylation. Methylation of the OXTR gene is reduced in AUD compared with healthy controls, with OT gene methylation being linked to craving and withdrawal severity, possibly via the interplay between the oxytocin system and the HPA‐axis. Our results suggest that future studies on the use of oxytocin as a therapeutic agent need to consider epigenetic regulation of its receptor and gene as a mechanism that could influence oxytocin's effect on craving and withdrawal symptoms.

## Author Contributions

PJP, MR, SB and TH designed the study and collected the data. Laboratory and statistical analysis were performed by PJP, MR and AFH. The manuscript was drafted by PJP and critically revised by MR, SB, TH, HF and AG. All authors read the final version of the article.

## Ethics Statement

The local Ethics Committee of the University of Erlangen‐Nuremberg approved the original study. The Ethics Committee at Hannover Medical School approved the described investigation (approval number 10399_BO_K_2022).

## Consent

All patients and participants gave written informed consent.

## Conflicts of Interest

The authors declare no conflicts of interest.

## Supporting information


**Figure S1.** Supporting Information

**Table S1:** Methylation details Oxytocin (OT)
Table S2: Methylation details (OXTR)
Figure S2: Age and Methylation for smokers and nonsmokers

**Data S1.** Supporting Information

**Data S2.** Supporting Information

## Data Availability

The data that support the findings of this study are available from the corresponding author upon reasonable request.
