# Supplementary figures and images for: The Role of Oxytocin and Oxytocin Gene Receptor Methylation During Withdrawal Therapy in Males With Alcohol Use Disorder
Source: Addict Biol. 2025 Jul 14;30(7):e70060. doi: 10.1111/adb.70060 (PMC12257889; doi:10.1111/adb.70060)

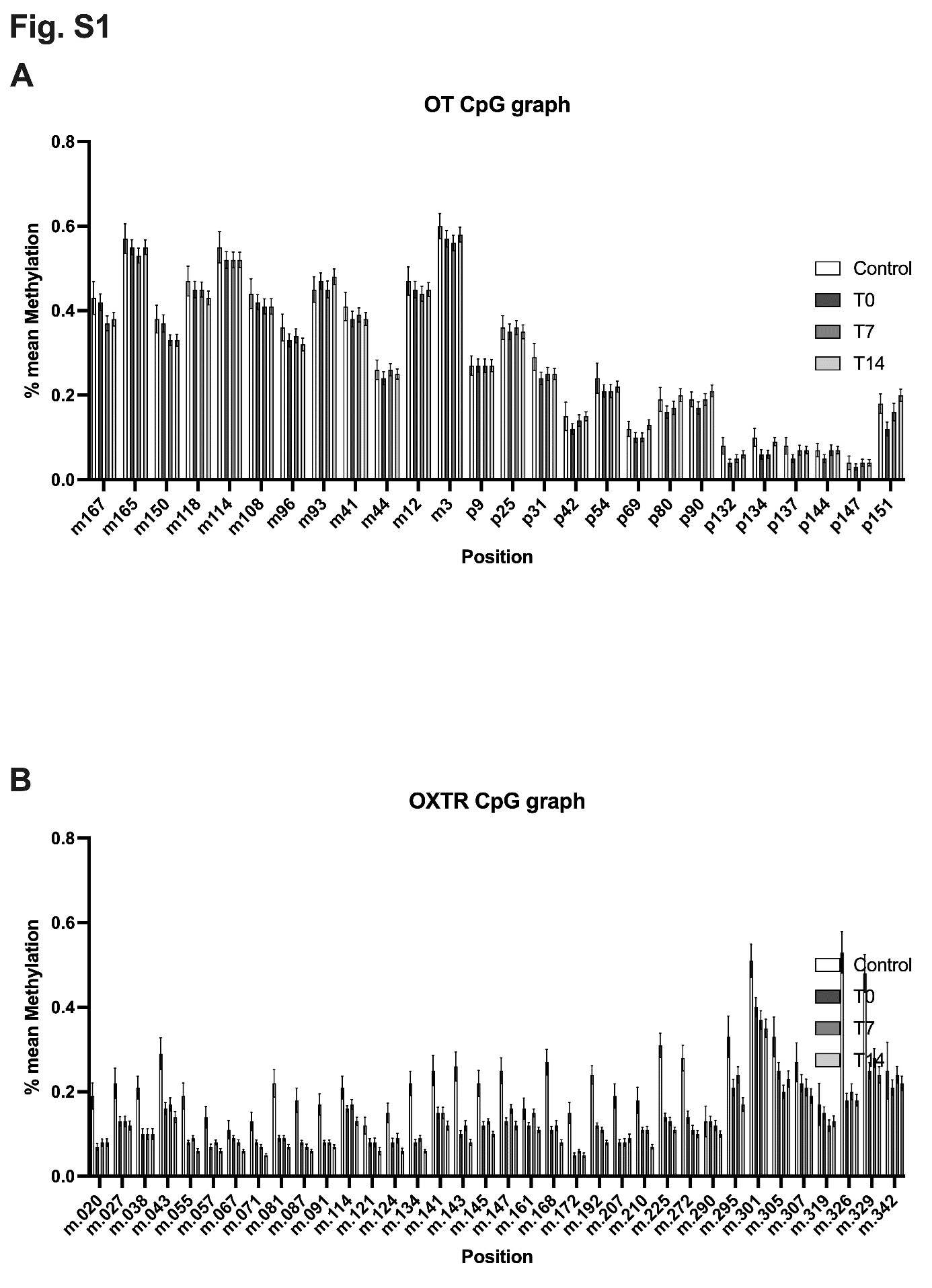

Supplement: Supplementary file 1 — Figure S1. Supporting Information [file ADB-30-e70060-s004.jpg]

Post-Hoc-Power Analysis

Oxyotcin Gene:


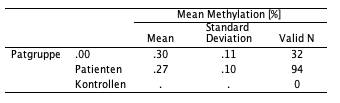


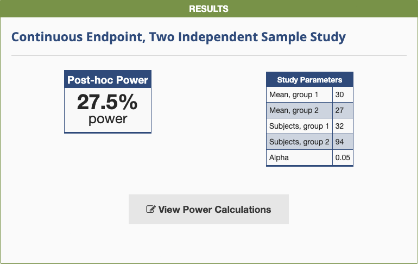


Oxytocin Receptor:


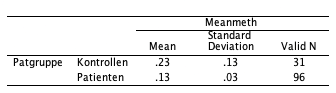


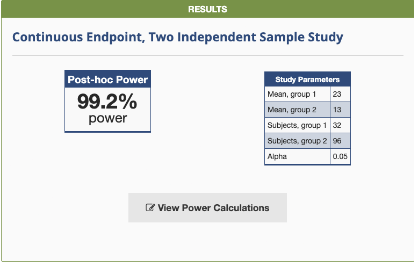

Supplement: Supplementary file 4 — Data S2. Supporting Information [file ADB-30-e70060-s003.docx]
