# Supplementary material for: The Role of Oxytocin and Oxytocin Gene Receptor Methylation During Withdrawal Therapy in Males With Alcohol Use Disorder
Source: Addict Biol. 2025 Jul 14;30(7):e70060. doi: 10.1111/adb.70060 (PMC12257889; doi:10.1111/adb.70060)

# Figure S2: Age and Methylation for smokers and nonsmokers

## OT graph


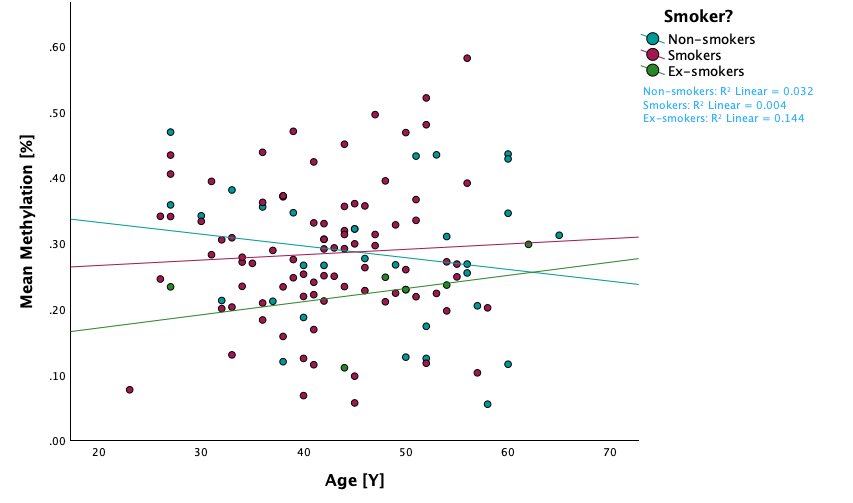


## OXTR graph


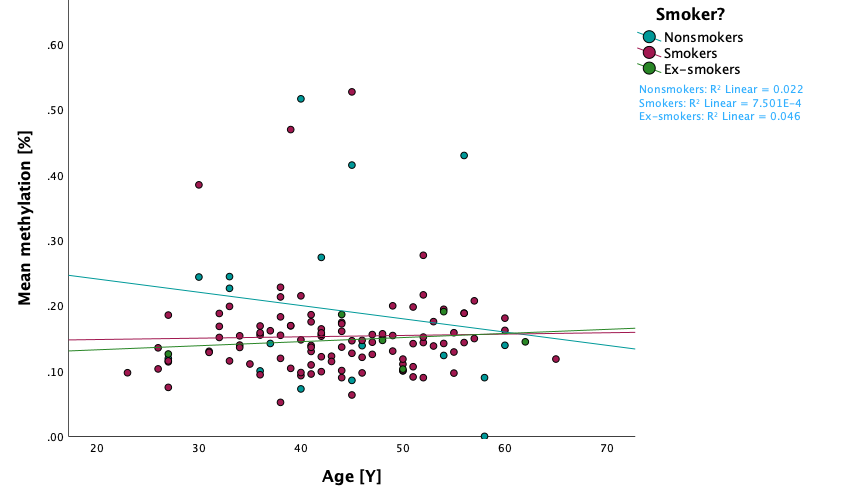


# Table S1: Methylation details Oxytocin (OT)


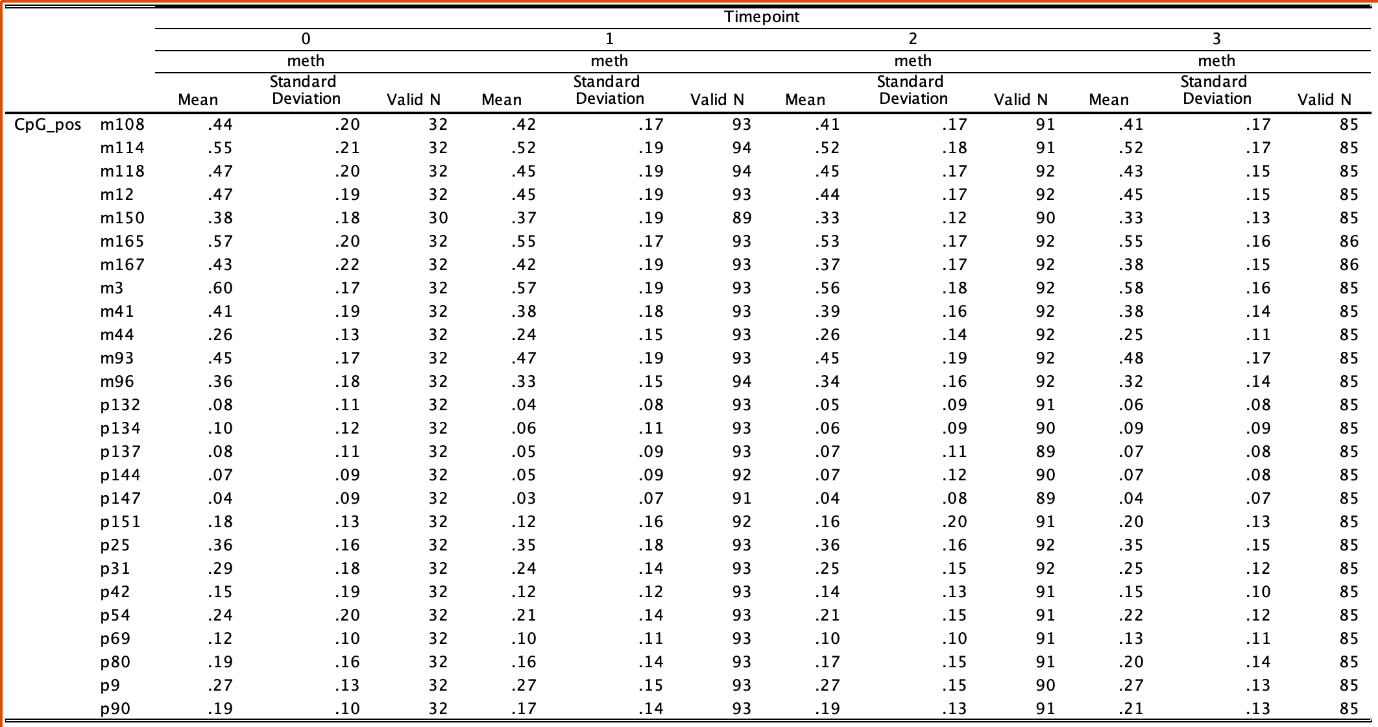


# Table S2: Methylation details (OXTR)


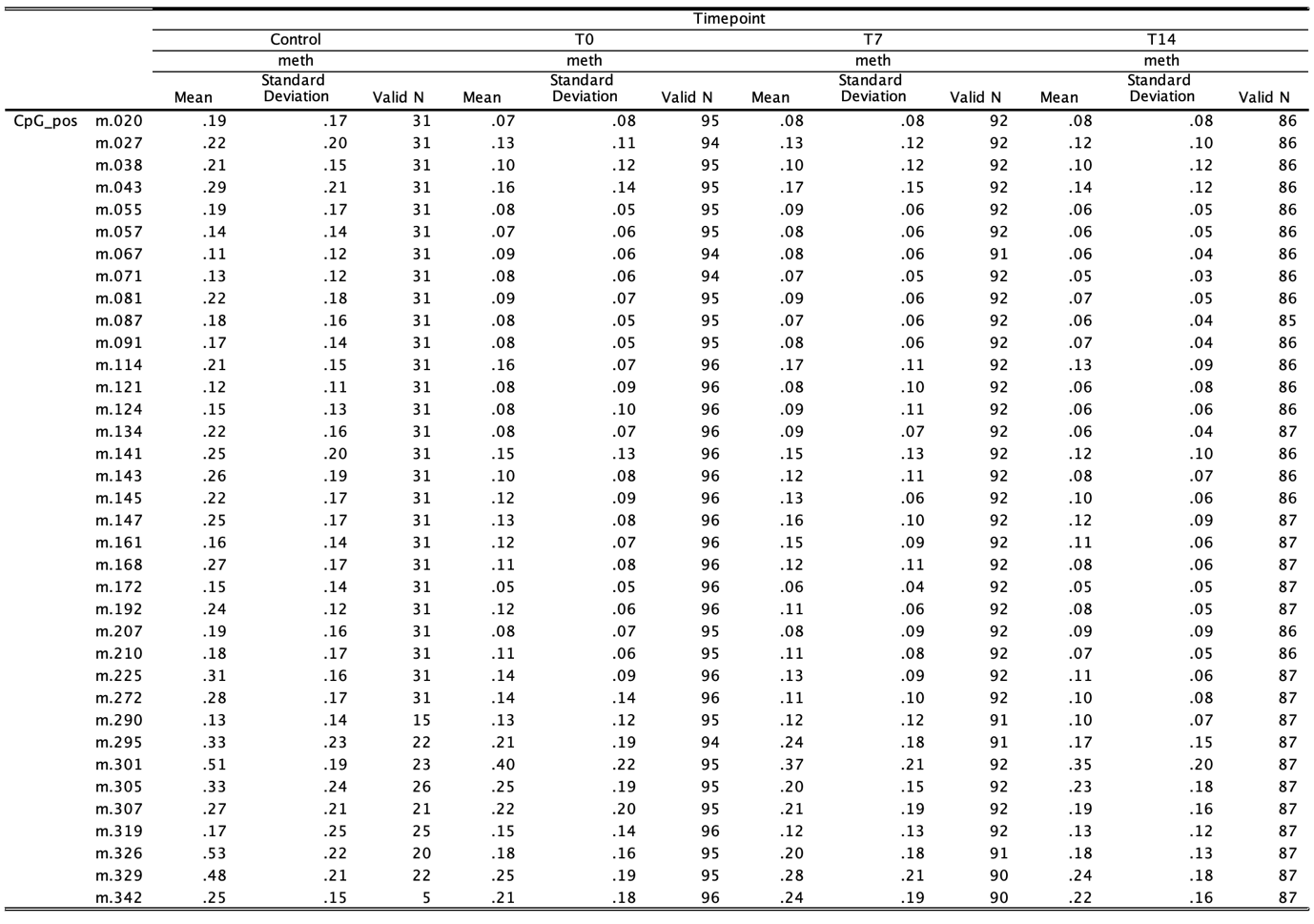

Supplement: Supplementary file 2 — Table S1: Methylation details Oxytocin (OT) Table S2: Methylation details (OXTR) Figure S2: Age and Methylation for smokers and nonsmokers [file ADB-30-e70060-s002.docx]
